# Thyroid carcinoma with discharging sinus – a rarity: a case report

**DOI:** 10.1186/1752-1947-2-64

**Published:** 2008-02-27

**Authors:** K Harish

**Affiliations:** 1 Department of Surgical Oncology, Gokula Curie Cancer Centre, M. S. Ramaiah Medical College & Hospital, Bangalore – 560054, India

## Abstract

**Introduction:**

Well differentiated thyroid carcinoma with isolated involvement of skin is extremely rare. Presentation with a discharging sinus has not been reported.

**Case presentation:**

This is a report of a 62 year old male patient with a long standing history of thyroid swelling which has metastasized to neck nodes and ulcerated over the midline resulting in a discharging sinus.

**Conclusion:**

Extra thyroidal extension is seen in 4% to 16% of all well differentiated thyroid cancers. However there is only an isolated report of skin ulceration over the thyroid. This is a unique case where the ulceration resulted in a discharging sinus. Patient is alive after 2 years following successful therapy. Aggressive surgery is warranted for locally advanced thyroid cancer.

## Introduction

Thyroid is enclosed in pretracheal fascia. Local invasion would usually involve the recurrent laryngeal nerve or the trachea. Involvement of skin is extremely rare more so when seen in isolation without tracheal or recurrent laryngeal nerve involvement. We report one such case with discharging sinus over the involved skin.

## Case presentation

**Figure 1:**
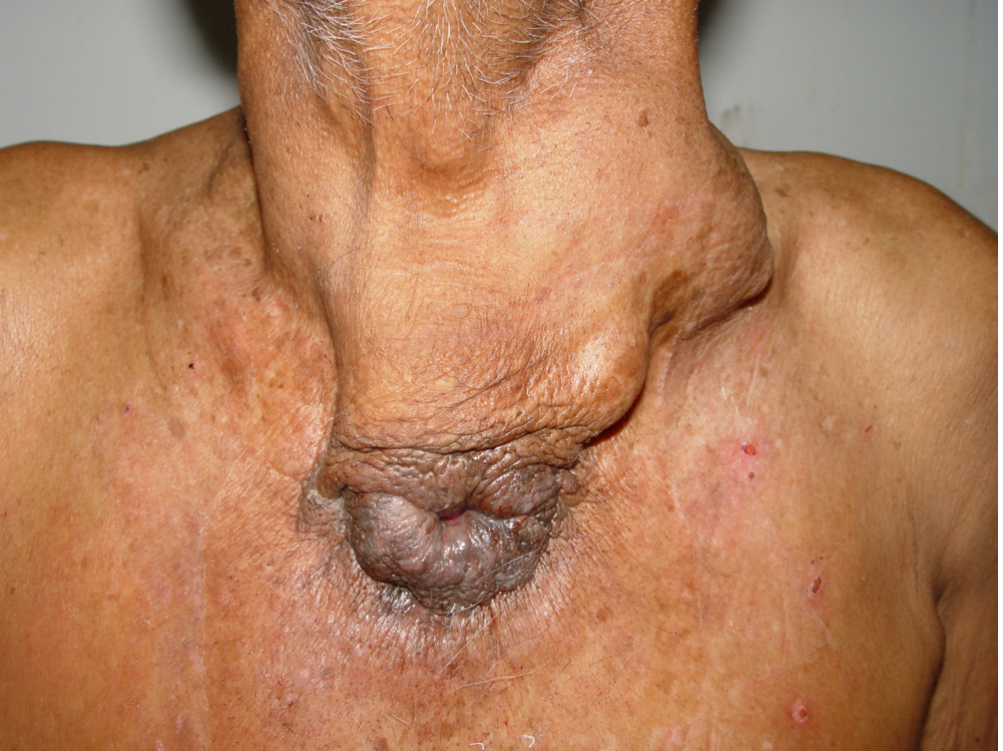
Photograph showing carcinoma of thyroid with sinus extending downwards and opening in front of the manubrium. The metastatic nodal disease on the left side of the neck can also be appreciated.

A 62 year old male patient presented to our hospital with a 9 year history of swelling in the neck. The swelling was situated in front of the neck in the thyroid region which gradually increased and 6 years later further swellings appeared in the left side of the neck. There were no other associated symptoms including those of hypo or hyperthyroidism. There was no difficulty in swallowing or breathing. Six months prior to presentation at our hospital, patient noticed a small ulceration in the skin over the tumor in the supra-sterna notch which did not heal (Figure 1). A small quantity of discharge from this sinus has persisted since then.

Examination revealed a swelling in the thyroid region, more prominent on the left side with multiple palpable nodes in the left neck. The skin surrounding the sinus was inflamed and thickened. Investigations revealed euthyroid status. There was neither infiltration of trachea nor retro-sternal extension on CT scan of the neck and upper chest. No contrast was used as it could interfere with post operative radio-iodine scans. There was no vocal cord palsy.

With a pre-operative diagnosis of papillary carcinoma of thyroid, patient underwent en-bloc total thyroidectomy including excision the sinus skin (2 cm margin) with strap muscles. In addition patient underwent a left modified radical neck dissection. It was possible to obtain primary closure of the skin incision. Post operative histopathology showed tall cell papillary carcinoma thyroid with infiltration of strap muscles and subcutaneous soft tissue mainly in the isthmus – left lobe junction. While the sinus showed malignancy, the skin was free of tumor (T4a, N1b, M0, Stage IVa). No metastases were detected on post operative radio-iodine scan. Patient underwent an ablative radioactive iodine scan post operatively and is on suppressive dose of thyroxine since then. In view of extensive extra-thyroidal spread, patient was administered 50 Gy of radiation over 25 fractions. Patient is on regular follow-up and is disease free for the last 2 years.

## Discussion

Papillary thyroid carcinomas constitute 80% to 85% of malignant epithelial thyroid tumors. Extra-thyroidal extension (ETE) occurs in 4% to 16% of cases and carries with it an increased risk of disease recurrence and death [1,2]. ETE does not represent a homogeneous group with a uniform prognosis [2]. Common ETEs include involvement of recurrent laryngeal nerve, larynx, trachea and esophagus. Involvement of skin with fixity is quite rare. Some findings emphasize the adverse impact of ETE in patients with differentiated thyroid cancer. ETE has a 54% 15 year survival and 29% 30 year survival [2]. In contrast, patients without ETE had a survival rate of 87% and a local failure rate of 9% at 30 years [2]. On the contrary there are reports that suggest 64% to 78% 15 year survival for complete versus 29% survival for incomplete resections [1,2]. Reports have shown that aggressive treatment for ETEs can result in long-term, disease-free survival [1,3,4]. Incomplete excision resulted in higher local failures while age more than 45 years predicted higher metastasis [2]. In this particular case there was ulceration resulting in a sinus but surprisingly with no nerve palsy or tracheal involvement. There are no large studies but a single report that address involvement of the overlying skin separately [5]. In that report, one was a medullary thyroid cancer and the other was a recurrent thyroid cancer post surgery and radiotherapy.

A number of aggressive variants of papillary thyroid carcinoma have been identified and are generally grouped as poorly differentiated. These include pathologic variants like tall cell, columnar, diffuse sclerosing and insular and carry poorer prognosis. Tall cell variant of papillary thyroid was described as an entity in 1976. Frequencies of soft-tissue involvement, tumor recurrence, and metastases were more common for tall cell variant of papillary carcinoma [6]. Surgical treatment is required, and resection of adjoining structures, including strap muscles and portions of the esophagus and trachea, may be necessary when involved.

The role of external radiation in the presence of presumed or microscopic disease is uncertain. There are evidences for and against such a usage although the data is entirely retrospective in nature.

## Conclusion

Well differentiated thyroid cancers should be addressed by aggressive surgery followed by radioactive iodine for ablation of residual disease. Surgery should address the primary disease, extra-thyroidal spread if any and nodal disease in addition. This surgery could include removal of structures like strap muscle. As an extension of the principle, structures that can be sacrificed like skin can also be removed for achieving optimal results. In conclusion, addressing the primary needs to be surgically aggressive more so when there is ETE in terms of skin involvement. External radiation should be considered in selected patients. Suppressive doses of thyroxine and regular follow-up would also be necessary.

## Abbreviations

ETE: Extrathyoridal extension.

## Competing interests

The authors declare that they have no competing interests.

## Authors' contributions

KH: Conceived the study, its design and coordination drafted the manuscript and approved the final manuscript.

## Consent

Written informed consent was obtained from the patient for publication of this case report and accompanying images. A copy of the written consent is available for review by the Editor-in-Chief of this journal.
